# Helicobacter Pylori infection among Iranian patients with normal upper gastrointestinal endoscopy

**DOI:** 10.22088/cjim.13.2.398

**Published:** 2022

**Authors:** Hassan Salmanroghani, Mohammad Hassan Rouzegari, Roham Salmanroghani

**Affiliations:** 1 Department of Internal Medicine, Shahid Sadoughi University of Medical Sciences, Yazd, Iran; 2 Department of Gastroenterology, Imam Khomeini Hospital Complex, Tehran University of Medical Sciences, Tehran, Iran

**Keywords:** Endoscopy, *Helicobacter pylori*, Gastric cancer, Dyspepsia

## Abstract

**Background:**

Helicobacter pylori infection can be a risk factor for non-cardia gastric cancer. In the present study, we aimed to assess the rate of *Helicobacter pylori* infection, its virulence factor and precancerous lesion among over 50 years old, dyspeptic patients with normal endoscopy.

**Methods:**

A total of 620 patients over 50 years of age with dyspepsia that referred to Shahid Sadoughi Hospital, Yazd, Iran from December 2018 to January 2019 were evaluated. One hundred fifty patients with normal appearance endoscopy were selected, and six gastric biopsy specimens were taken from each subject. Data were analyzed using chi-square and logistic regression tests.

**Results:**

A total of 150 patients with mean age of 65.8±11.9 years old participated in this study. Sixty-three (42%) patients were males. Thirty-four (22.6%) patients had precancerous lesions. Ninety (60%) patients had positive PCR results for *H. pylori*. *H. pylori* infection test was positive in 24 (70.6%) patients with precancerous lesion. Sixty-six (57%) patients without the precancerous lesions (116 cases) were positive for *H. pylori.*

**Conclusion:**

There was no a significant difference in the rate of *H. pylori* infection and its genotype distribution was between patients with and without the precancerous lesions.

Helicobacter pylori (*H. pylori*), or classically called Campylobacter pyloridis, is found in about 50% of peoples all around the world. *H. pylori* has been proposed to be causative in gastrointestinal disorders, such as gastritis, peptic ulcer, gastric cancer (GC) or mucosa-associated lymphoid-tissue lymphoma (1). Stomach cancer is the most common cancer in men and is the third cancer after breast and colorectal cancer among women in Iran (2). The International Agency for Research on Cancer (IARC) has classified *H. pylori* as the group 1 carcinogen since 1994 (3). The previous studies have revealed that the occurrence of gastric cancer has increased in populations with high prevalence of *H. pylori* infection. Prevalence of *H. pylori* infection is high among the Iranian population and the age of acquisition of infection in Iran is low (4). *H. pylori* virulent factors are important when combined with host and environmental factors (5). Researchers have reported that *H. pylori* infection, although is closely linked to the development of gastric cancer, does not increase all-cause mortality. (6). Significant variations in vacuolating activities are observed between the different strains of H.* pylori*. These variations are attributed to the polymorphisms of *vacA* gene that play a key role in vacuolating activity and is significantly linked to gastric adenocarcinoma. The *vacA-d1 type* gene is associated with neutrophil infiltration and gastric mucosal atrophy in both the antrum and the corpus.

It seems that the genotype of d region is an important risk locus for gastric cancer in Western strains. The cytotoxin-associated gene A (*cagA*) is another virulence factor of *H. pylori* which is more likely to have a significant role in the development of gastric cancer (7-9). The prevalence of stomach cancer is high in the North and the Northwestern province and low in the South and the Southeastern province of Iran. It seems that the prevalence of GC in Yazd is in the middle of this spectrum (10). The two important factors that increase the chances of getting gastric cancer are *H. pylori* infection specially infection with more virulent subtypes and existence of precancerous lesions. Evaluation of these two important factors in the high-risk group may help us for the better management of this malignancy. In the present study, we aimed to assess the prevalence of *H. pylori* infection; its virulent antigen and the precancerous lesions among patients with dyspepsia that referred to Shahid Sadoughi Hospital, Yazd, Iran.

## Methods

**Patients: **A total of 620 patients over 50 years of age with dyspepsia that referred to Shahid Sadoughi Hospital, Yazd, Iran (from December 2018 to January 2019) were entered to the study. One hundred-fifty patients with normal endoscopy were selected (by Census Method) (11) and its data, including demographic, information, familial and past medical histories were collected by a questionnaire. Inclusion criteria were age above 50 years and having normal endoscopic appearance results. Certain exclusion criterion was the use of antibiotic within the last 1 month. This study was approved by the Ethics Committee of Shahid Sadoughi University of Medical Sciences-Yazd (IR.SSU.MEDICINE.REC.1395.248).

**Gastric Biopsy: **Six gastric biopsy specimens were taken from each subject. Samples included two biopsies from incisura angularis for urease assay and PCR test, two biopsies from the antrum, two biopsies from the corpus at greater and lesser curvature for histologic evaluation (12). The biopsies were collected by pathologists and immediately were placed into neutral buffered formalin. The number and site of the biopsies were registered for each patient. The histological sections were examined by two experienced gastrointestinal pathologists.

**DNA extraction, detection and genotyping of** ***H. pylori*** **: **Samples from the incisura angularis of the stomach were sent to immunology research center in Tehran University of Medical Sciences for PCR amplification. DNA extractions were performed (by German Roche Co. kit) according to the Roche DNA extraction kit instruction (13). Polymerase chain reaction (PCR) tests were performed using the primers summarized in table 1, and the different genotypes of *H. pylori* were detected. We used a positive control and a negative control in the PCR reaction.

**Table 1:** Oligonucleotide primers used for PCR

**GENES**	**Primers**	**Sequence**	**Size of PCR products (bp)**	**Annealing Temperature (°C)**	**Reference**
16S rDNA	HP1	GCAATCAGCGTCAGTAATGTTC	519	56	(29)
	HP2	GCTAAGAGATCAGCCTATGTCC			
vacA	s1a-F	GTCAGCATCACACCGCAAC	190	54	(30)
	s1a-R	CTGCTTGAATGCGCCAAAC			
	s1b-F	AGCGCCATACCGCAAGAG	187	54	(30)
	s1b-R	CTG CTT GAA TGC GCC AAA C			
	s2-F	GCT AAC ACG CCA AAT GAT CC	199	54	(31)
	s2-R	CTG CTT GAA TGC GCC AAA C			
	VAG-F	CAA TCT GTC CAA TCA AGC GAG GCG	570	56	(31)
	VAG-R	GCG TCT AAA TAA TTC CAA GG			
	VAC F1	GTTGGGATTGGGGGAATGCCG	426	53	(31)
	C1R	TTAATTTAACGCTGTTTGAAG			
	VACF2	GTTGGGATTGGGGGAATGCCG	432	53	(31)
	C2R	GATCAACGCTCTGATTTGA			
	VAS-5 F	ACTAATATTGGCACACTGGATTTG	d1: 367–379	45	(31)
	VAGF-R	CTCGCTTGATTGGACAGATTG	d2: 298		
	mD1-F	AGGTYATTAACCCACCCAA	d1: 223	45	
	md1-R	CTCGCTTGATTGGACAGATTG			
cagA	cagAN-F	CCATTTTAAGCAACTCCATAAACC	413	56	(31)
	cagAN-R	CTGCAAAAGATTGTTTGGCAGA			
	cagAC-F	GGCAATGGTGGTCCTGGAGCTAGGC	243	55	(31)
	cagAC-R	GGAAATCTTTAATCTCAGTTCGG			
	CAG1	ACC CTAGTC GGT AAT GGG TTA	591-856	50	(31)
	CAG2	GTA ATT GTC TAG TTT CGC			

***Histopathologic Examination: *** Biopsies were stained with Giemsa for *H. pylori *and checked for the precancerous lesion (atrophic gastritis or intestinal metaplasia). Histopathological evaluations were performed based on the updated Sydney classification system and were divided into two groups; group one (with precancerous lesion (atrophic gastritis or intestinal metaplasia) and group two (without the precancerous lesion) (14). Gastric mucosal atrophy is defined as the loss of mucosal glands and a reduction in the mucosal thickness that is usually associated by increasing the stromal matrix. Intestinal metaplasia (IM) is defined as the replacement of gastric mucinous epithelial cells with small intestinal cells such as goblet cells. The presence of these cells is the minimum diagnostic criteria for intestinal metaplasia (17).

 In order to histologic findings, patients were classified into two groups: groups I with positive histologic findings and groups II without histologic findings.

**Data analysis: **Data were analyzed using chi-square and logistic regression model tests in SPSS software Version 19 (SPSS Inc. Chicago, IL). A *p-*value less than 0.05 was considered a statistically significant level.

## Results

A total of 150 patients (mean age: 65.8±11.9) were enrolled to the study. Sixty-three (42%) patients were males and 87(58%) cases were females. Thirty-four (22.6%) patients had precancerous lesions. 17 (50%) patients are in group 1 (with atrophic gastritis or intestinal metaplasia), 46 (40%) patients in group 2 (without precancerous lesion) were males, while 70 (60%) patients were females (table 2). Rapid urease test was positive in 63% of patients. 

**Figure 1:**
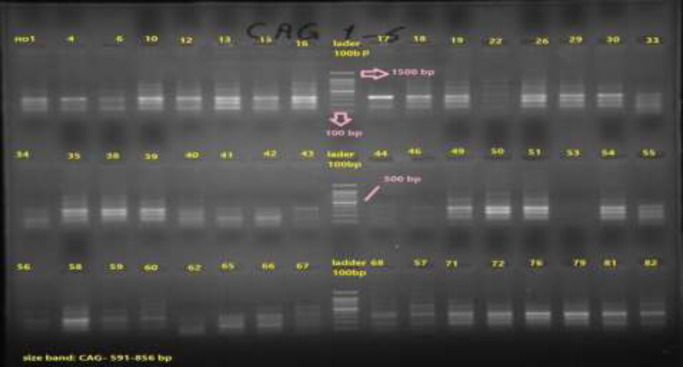
PCR product of CAG A gene on the gel electrophoresis

*H. pylori* infection was detected using the PCR test (fig 1). Ninety (60%) patients had positive PCR results for *H. pylori*. Twenty-four patients with PCR positive test had precancerous lesions. Nine patients in group I had positive results for *vac A S1* and 12 patients were positive for *vac A S2*. Twenty patients of group II (without precancerous lesion) had positive results for *vac A S1* and 39 patients were positive for *vac A S2*. The p-value was not statistically significant (p-value > 0.05) (table 3). Five patients in group I had positive results for *vac A M1* and 3 patients were positive for *vac A M2*. In group II, 17 of 66 patients had positive results for vac A M1 and 11 of 66 patients had positive results for vac A M2. In terms of vac A C1, 13 of 24 patients had positive results in group I and 31 of 66 patients had positive results in group II. In addition, in group I, 13 of 24 patients had positive results for vac A C2 and 38 of 66 patients had positive results in group II. In precancerous group, 12 of 24 patients had positive results for vac A i1 and 16 of 24 patients had positive results for vac A i2. In group two (without precancerous lesion), 39 of 66 patients had positive findings for vac A i1 and 46 of 66 patients had positive findings for vac A i2. In group I, 2 of 24 patients had positive findings for vac A d1 and 3 of 24 patients had positive findings for vac A d2. In group II, 8 of 66 patients had positive results for vac A d1 and 2 of 66 patients had positive results for vac A d2 (p>0.05) (table 3). In the precancerous group, 21 of 24 patients had positive results for Cag A and 3 of 24 patients had negative results for Cag A. In group II (without precancerous lesion), 50 of 66 patients had positive results for Cag A and 16 patients of 66 patients had negative results for Cag A (p>0.05).

**Table 2:** Gender and Frequency of H. pylori in different Groups

		**Group One**	**Group Two**	**Total**
Gender	Male	17 (50)	46 (40)	63(42)
	Female	17 (50)	70 (60)	87(58)
*H. pylori* PCR	16 S rDNA +	24 (70.6)	66 (57)	90(60)
	16 S rDNA -	10 (29.4)	50 (43)	60(40)

**Table 3:** Logistic Regression of H. pylori genotypes in Different Groups

**Genotype **	**Group One** **(with pre.CA) N=24** **N (%)**	**Group Two** **(without pre.CA) N=66** **N (%)**	**Total** **N (%)**	**P-value**	**OR**	**95% CI**
Cag A +	21 (87.5)	50 (75.7)	71 (78.8)	0.23	2.24	0.59-8.50
Vac A S1M1	3 (12.5)	4 (6.06)	7 (7.7)	0.323	2.21	0.45-10.71
Vac A S1	9 (37.5)	20 (30.3)	29(32.2)	0.51	1.38	0.51-3.67
Vac A S2	12 (50)	39 (59.09)	51 (56.6)	0.44	0.69	0. 27-1.77
Vac A M1	5 (20.83)	17 (25.75)	22 (24.4)	0.63	0.75	0.24-2.34
Vac A M2	3 (12.5)	11 (16.6)	14 (15.5)	0.63	0.71	0.18-2.81
Vac A C1	13 (54.1)	31 (46.9)	44 (48.8)	0.54	1.33	0. 52-3.40
Vac A C2	13 (54.1)	38 (57.57)	51(56.6)	0.77	0.87	0. 34-2.22
Vac A i1	12 (50)	39 (59.09)	51 (56.6)	0.44	0.69	0.27-1.77
Vac A i2	16 (66.69)	46 (69.69)	62 (68.8)	0.78	1.15	0.42-3.12
Vac A d1	2 (8.3)	8 (12.12)	10 (11.11)	0.61	0.65	0.13-3.34
Vac A d2	3 (12.5)	2 (3.03)	5 (5.5)	0.10	0.21	0.03-1.39

## Discussion

The effect of *H. pylori* on GI tract is different due to the host and microbial factors. The site of gastritis caused by *H. pylori* determines the outcomes. If corpus predominant atrophic gastritis is present, it can progress to both gastric ulcer or intestinal metaplasia. Finally, intestinal metaplasia can progress to dysplasia and gastric cancer (15). The prevalence of precancerous lesions in the current study was 22%. In addition, the prevalence of *H. pylori* was 60%. The incidence of GC is low in our area (YAZD) compared to other cities in the North and Northwestern of Iran. The incidence of gastric cancer is 12.84 per 100000 in men and 7.35 per 100000 in women. The exact worldwide prevalence of gastric precancerous lesions such as intestinal metaplasia is unknown. A positive association has been obtained between precancerous lesions and GC(16). A retrospective study performed by Sonnenberg et al. in the United States reviewed 78985 patients who underwent upper endoscopy. The prevalence of intestinal metaplasia was 7%. The presence of H pylori, chronic active gastritis and intestinal metaplasia was strongly associated with each other. In the USA, gastric cancer incidence is 2.8 per 100000 in men and 1.5 per 100000 in women (17). The prevalence of *H. pylori* in 938 people (age 17 to 26 years old) entering the US Army was 26.3%. This prevalence is stabilized over time in the USA (18).

The highest incidence of gastric cancer in Iran has been reported from Ardabil with an ASR of 49.1 and 25.4 in men and women, respectively. The overall prevalence of precancerous lesions and *H. pylori* infection in Ardebil is 54% and 89%, respectively based on the previous reports (19). Therefore, it seems that, the *H. pylori* infection can be a significant risk factor in the incidence of gastric cancer, especially in susceptible persons who developed precancerous lesions. Different *H .pylori* infection rates and other environmental factors can explain the intercountry variation of gastric cancer prevalence (20). A meta-analysis study, including seven randomized investigations revealed that the eradication of *H. pylori* can decrease the risk of gastric cancer by 35% (21). Other risk factors, such as dietary and smoking may contribute to the development of stomach cancer in an inflammatory background caused by this bacterium. 

There are a lot of controversies about the carcinogenicity of different *H. pylori* strains. Genetic studies have demonstrated that *vac A *may be associated with peptic ulcers, severe gastritis and both precancerous and cancerous lesions. It has different polymorphic regions which have shown to have contributed many gastroduodenal disorders (22).

In several studies, the prevalence of some subtypes of *H. pylori* has shown to be more frequent in precancerous lesions and gastric cancer than those without precancerous lesions. (23, 24). A meta-analysis study conducted on 17374 patients showed that *H. pylori* genotypes *vacA s1* (vs. s2), m1 (vs. m2) and s1m1 (vs. s1m2 or s2m2) are associated with the increasing risk of development of gastric cancer (25). In our study, the prevalence of *vacA s1* was higher in the precancerous group than the group without precancerous lesions, but *vacA m1* was higher in the group without precancerous lesions with the odds ratio of 1.380 (CI: 0.518-3.673) and 0.759 (CI: 0.245-2.346), respectively. The prevalence of *vacA s1m1* was in favor of precancerous lesions versus the group without precancerous lesions with the odds ratio of 2.214 (CI: 0.458-10.714). The prevalence of *vacA S2*, M2, was higher in the group without precancerous lesions than the precancerous lesion group, with odds ratio of 0.692 (CI: 0. 271-1.770) and 0.714 (CI: 0.181-2.817), respectively. 

In another study conducted by Bakhti et al. in Ardebil, the prevalence of genotype *vac A* *C1* (vs. C2) was significantly higher in the gastric cancer group than the non-atrophic gastritis group (26). In our study, the prevalence of *vac A c1* was higher in the precancerous group than the group without precancerous lesions with odds ratio of 1.334 (CI: 0.523-3.406) and the prevalence of C2 was higher in the group without precancerous lesions than the precancerous lesion group, with odds ratio of 0.871 (CI: 0. 340-2.229).

*Cag A* is one of the most important factors in the evolution of precancerous lesions. It is introduced into the host cells by encoding Cag-PAI with *H. pylori*, leading to further activation of cellular pathways and cell injury (8). In our study, *Cag A* was positive in 71 of 90 patients and not found significantly higher in precancerous lesions than the group without precancerous lesions, but in a logistic regression test, the odd ratio for *Cag A* was 2.240 (CI: 0.590-8.506) in favor of precancerous lesions versus the group without precancerous lesions. Another study revealed that most of the *H. pylori* strain in Iran are Cag positive (27).

In a study performed by Latifi-Navid et al., the prevalence of different genotypes of *H. pylori* was analyzed in ten cities of Iran. They divided these cities into two categories (high and low GC incidence), and estimated the prevalence of the different genotypes of *H. pylori* in these groups. Their results showed no significant difference regarding the distribution of *vac A s1, s2, m1* and *m2* genotypes and *iceA1, iceA2, babA2* and *cag A* genes among *H. pylori* isolates from areas with high and low incidence GC rates. They revealed a higher prevalence of the *vac A* *d1* and* i1* genotypes and *iceA1/2* gene among *H. pylori* isolates from the areas with high incidence GC rates in comparison with those in the low incidence areas and a higher prevalence of the *vac A* *d2* and *i2* in the low incidence GC areas in Iran (28). In our study, the prevalence of the *vac A d1, i1* and *i2* genotypes was higher in the group without precancerous lesions than the precancerous pathology group while *vac A d2* was higher in the precancerous pathology group. Our findings between the two groups (with and without the precancerous lesions) related to different genotypes of *H. pylori* were not statistically significant. 

In conclusion there was not a significant difference in the rate of *H. pylori* infection and its genotype distribution between the patients with and without the precancerous lesions.
